# Supplementary figures and images for: Dominance modifiers at the Arabidopsis self-incompatibility locus retain proto-miRNA features and act through non-canonical pathways
Source: PLoS Genet. 2026 Apr 20;22(4):e1012127. doi: 10.1371/journal.pgen.1012127 (PMC13128112; doi:10.1371/journal.pgen.1012127)

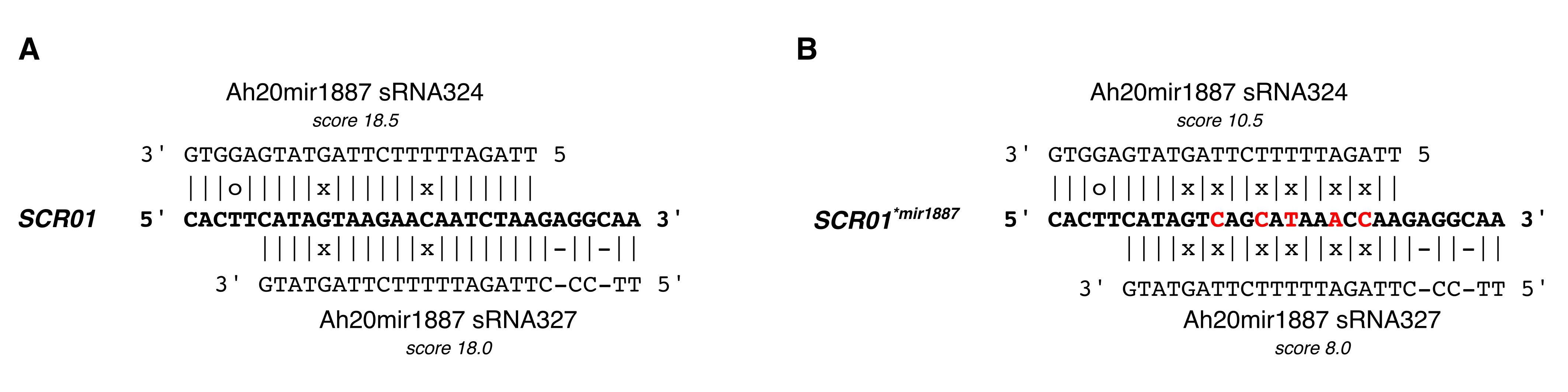

Supplement: S2 Fig — (A) Alignment between the native mir1887 target site on SCR01 and Ah20mir1887 sRNAs with a targeting score ≥ 18. (B) Alignment between the same sRNAs and the mutated mir1887 target site of the transgenic line SCR01*mir1887. Note that the mutations decrease the homology score below 18, suggesting that these sRNAs are not able to target the mutated site. Mutated sites are in red. (TIFF) [file pgen.1012127.s008.tiff]

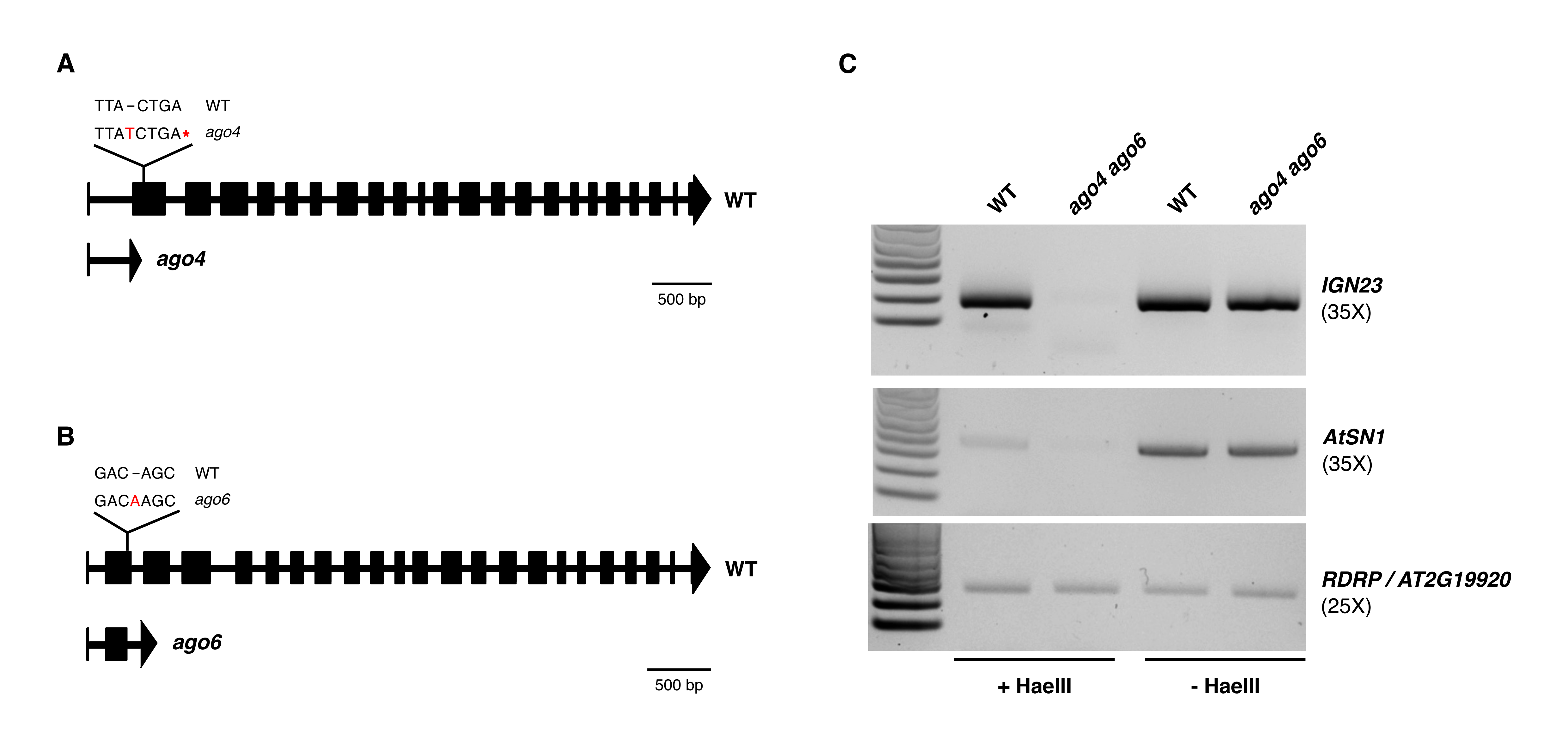

Supplement: S3 Fig — (A-B) Schematic representation of CRISPR/Cas9 induced mutations in the AGO4 and AGO6 genes. Sequence changes are shown in red, premature stop codons are represented by the asterisk. (C) Chop-PCR assay on WT C24 plants and ago4 ago6 double mutant plants. IGN23 and AtSN1 correspond to RdDM-dependent methylated regions, while RDRP corresponds to a non-methylated region. (TIFF) [file pgen.1012127.s009.tiff]

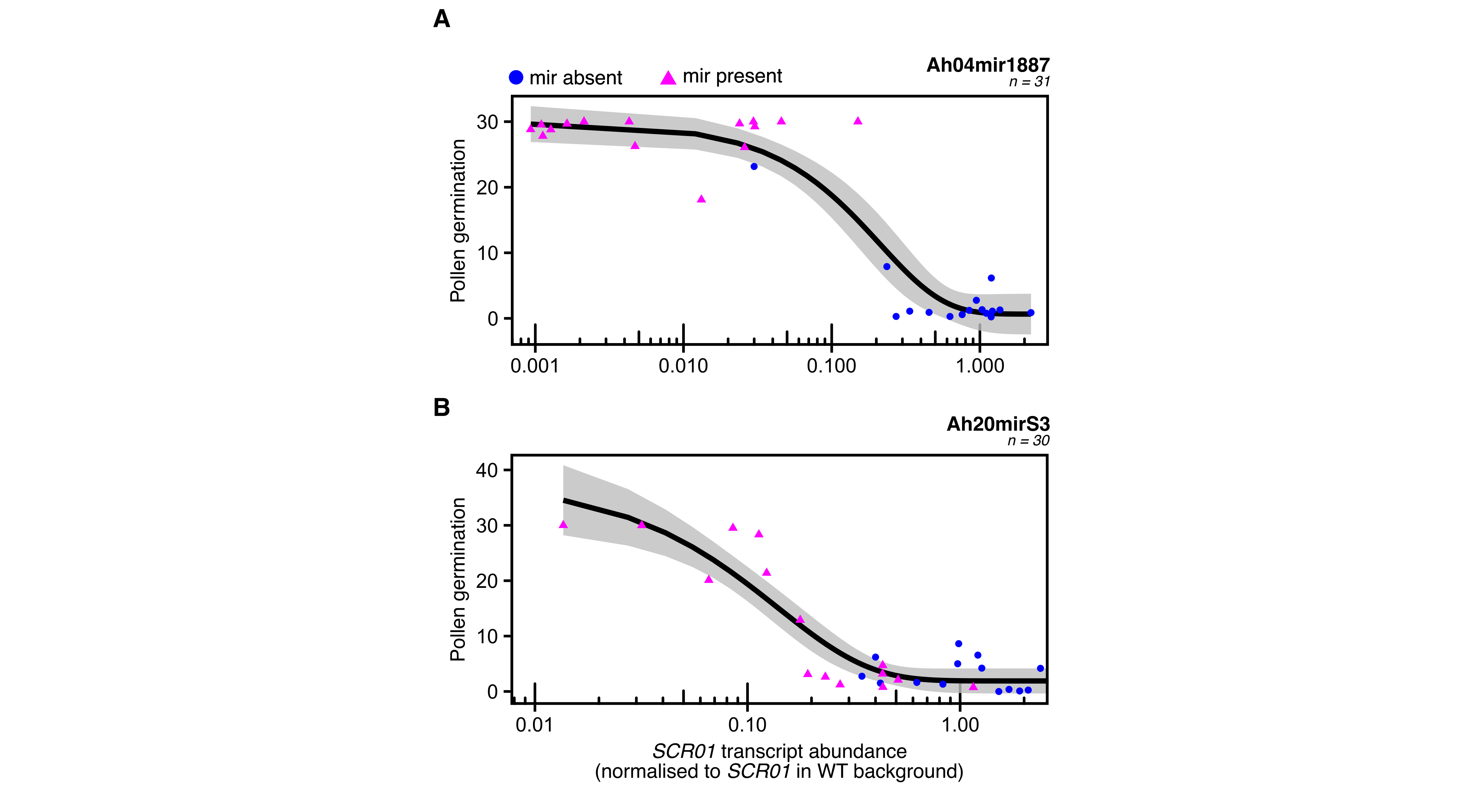

Supplement: S4 Fig — Non-linear regression analysis modeling the relationship between SCR01 transcript abundance and pollen germination. For both Ah04mir1887 (A) and Ah20mirS3 (B) sRNA precursors, a compatible pollen germination phenotype (~20–30 germinated pollen grains) requires approximately a 10-fold decrease in SCR01 transcript abundance compared to wild-type levels. Modelling was performed using the SSasymp self-starting asymptotic regression model in R. This analysis was based on the phenotypic and expression data presented in Fig 1C and 1D. SCR01 expression was measured once in each pollen donor, while pollen germination was recorded on multiple pistils pollinated with the same pollen donor. To account for this non-independence, germination values were averaged per plant prior to model fitting. The black line represents the predicted model, and the gray area corresponds to the 95% confidence interval. (TIFF) [file pgen.1012127.s010.tiff]

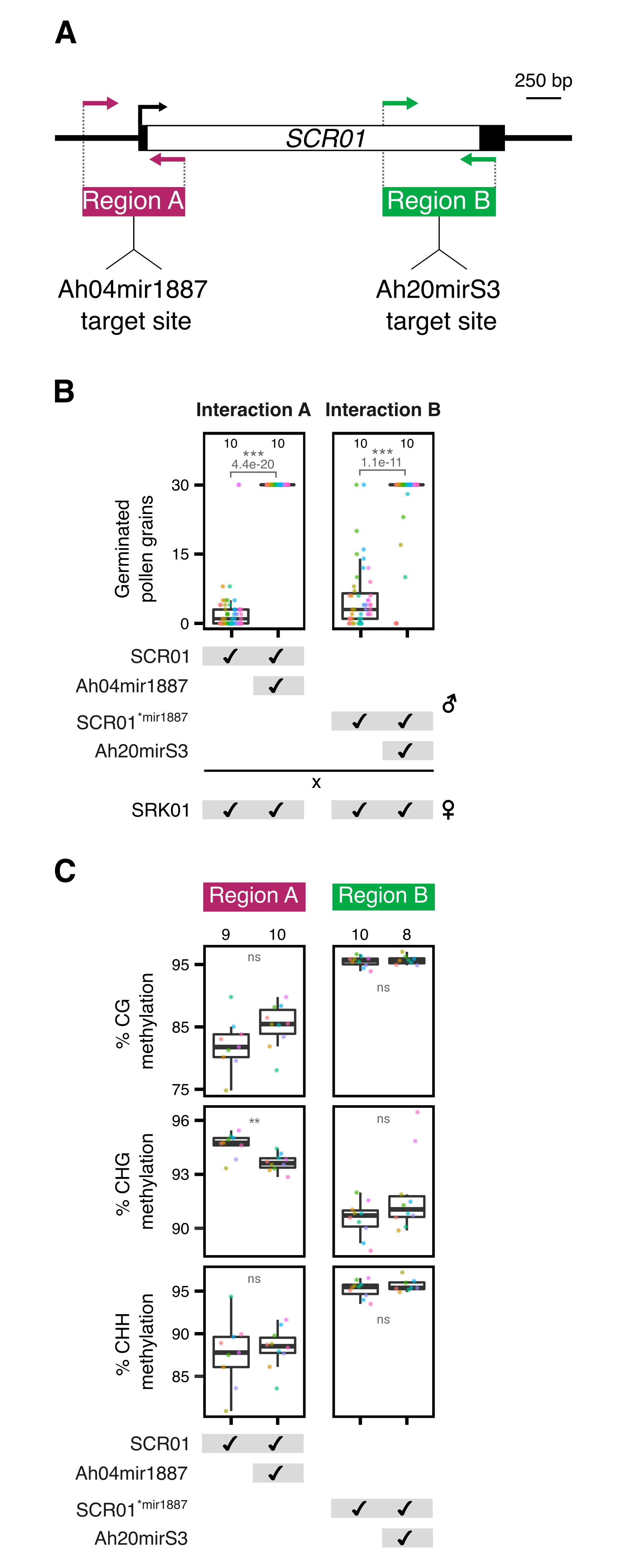

Supplement: S5 Fig — (A) Schematic representation of the BSAS assay performed to measure DNA methylation at the Ah04mir1887 target site (SCR01 5’ region - Region A), and at the Ah20mirS3 target site (SCR01 intron - Region B). Region A and Region B correspond to fragments of 794 and 871 bp, respectively, centered in each sRNA precursor’s target site. (B) Phenotypic validation of lines used for the BSAS assay. Colored dots correspond to values obtained for each biological replicate. Number of biological replicates is indicated at the top of the panel. For each biological replicate pollen germination on a total of 5 SRK01 pistils was counted. Statistical differences were assessed using a two-tailed Mann-Whitney test with continuity correction (C) Average DNA methylation levels on Region A (Interaction A) and Region B (Interaction B) of SCR01. DNA methylation levels correspond to the average of methylation in all cytosines in the top and bottom DNA strands of sampled regions. Measurements are paired, with each plant used for both pollination (panel B) and the BSAS experiment (panel C) represented by the same color dot, allowing a direct comparison of phenotypic response and DNA methylation levels for each individual. The number of biological replicates is indicated at the top of the panel. Statistical differences were assessed using a two-tailed Mann-Whitney test with continuity correction (ns - non significant, p-value > 0.05; ** - p-value ≤ 0.01). (TIFF) [file pgen.1012127.s011.tiff]

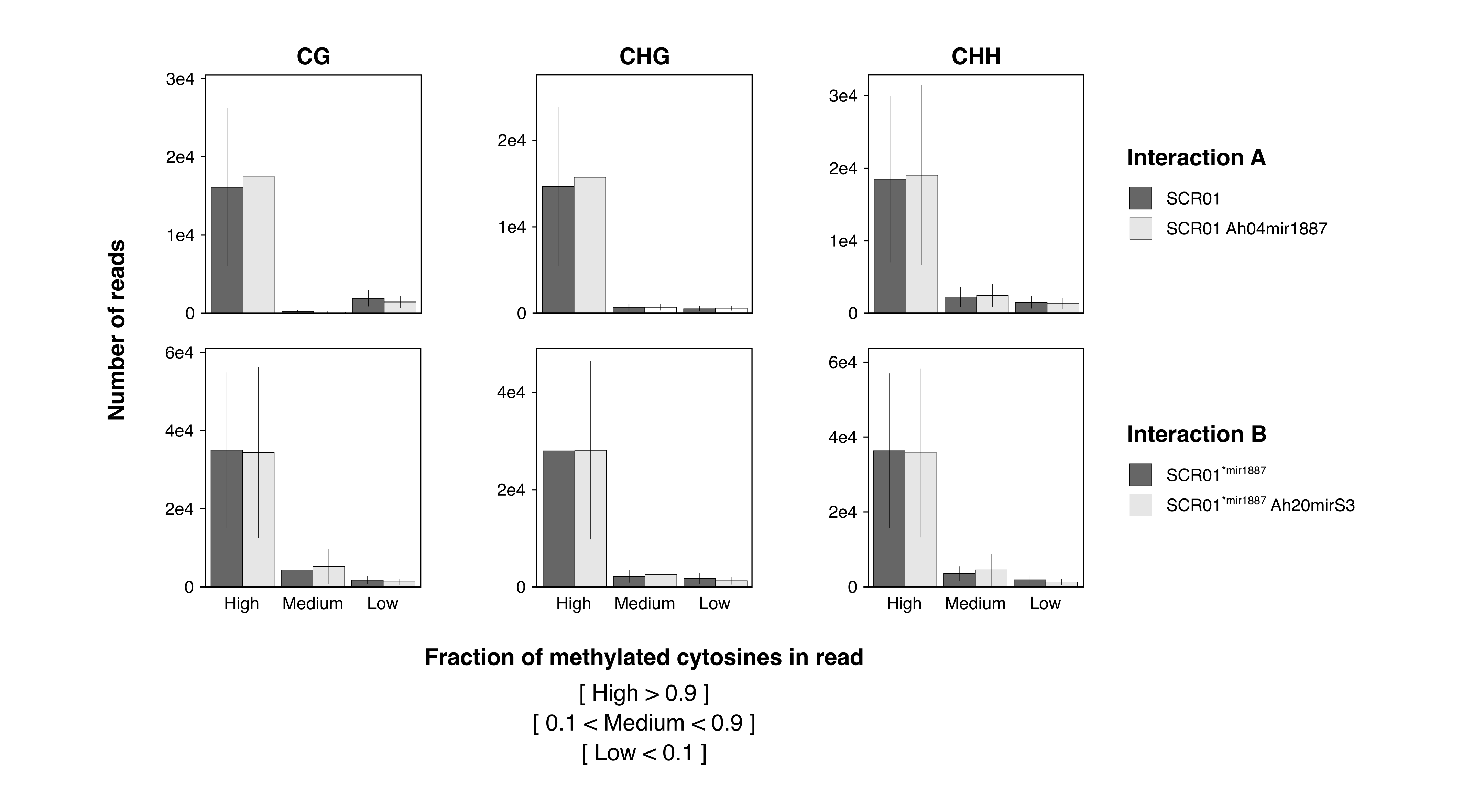

Supplement: S6 Fig — BSAS reads were grouped according to the fraction of methylated cytosines within each read. High: reads where nearly all cytosine positions are methylated (> 90% cytosines); Low: reads where very few cytosine positions are methylated (< 10% cytosines); Medium: reads with more than 10% and less that 90% cytosines methylated. Error bars represent the standard deviation between replicates. No statistically significant differences were detected in the number of highly, medium and lowly methylated reads between the SCR01 and SCR01 Ah04mir1887 or SCR01*mir1887 and SCR01*mir1887 Ah20mirS3 genotypes (p-value > 0. 05, two-tailed Mann-Whitney test with continuity correction). (TIFF) [file pgen.1012127.s012.tiff]

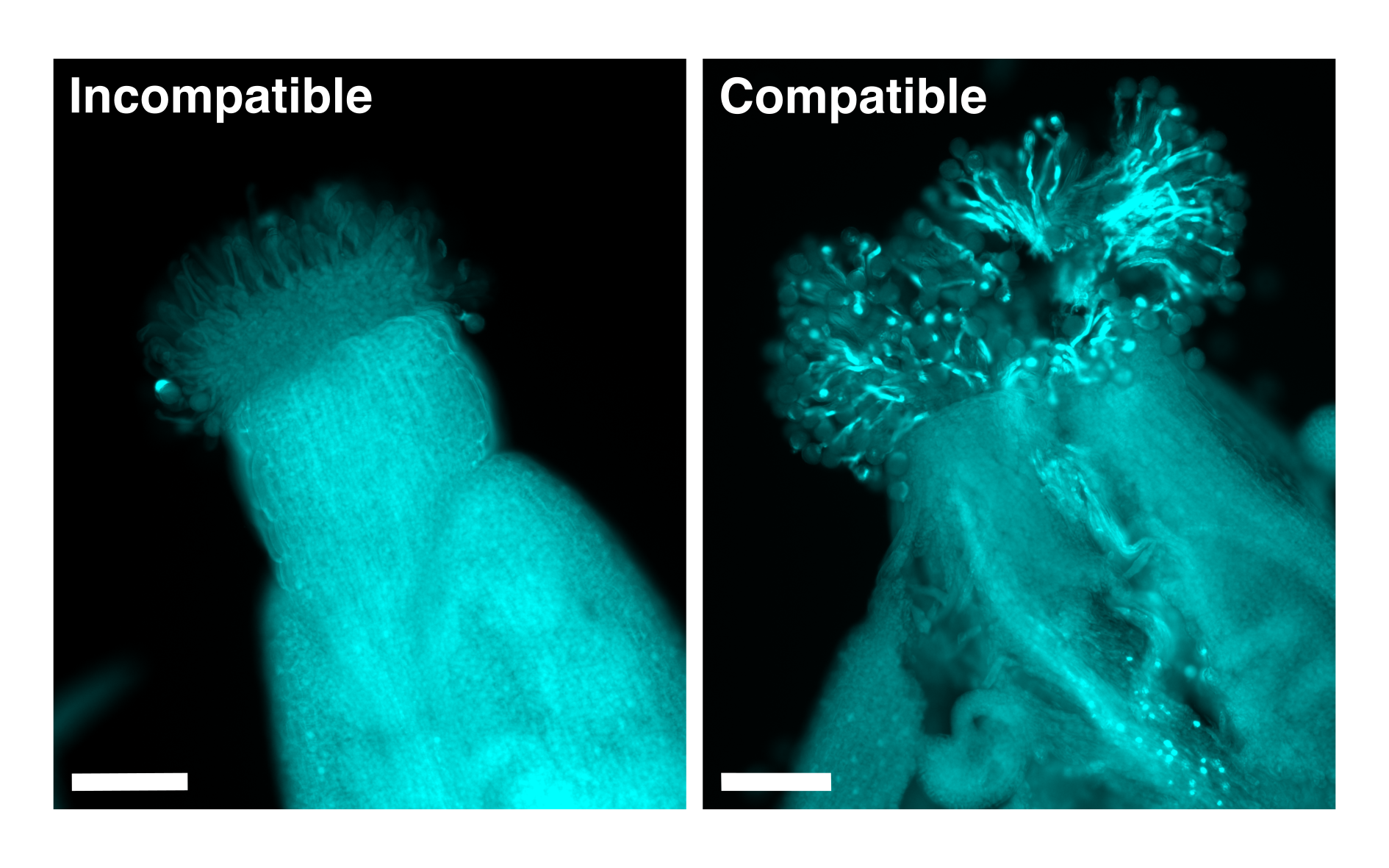

Supplement: S7 Fig — Pistils were pollinated with incompatible (left) or compatible (right) pollen and stained with aniline blue. The right panel shows strong fluorescence corresponding to germinating and growing pollen tubes within the style, characteristic of a compatible pollen reaction. In contrast, incompatible pollen (left) fails to germinate or produce elongating pollen tubes. Scale bar: 100μm. (TIFF) [file pgen.1012127.s013.tiff]

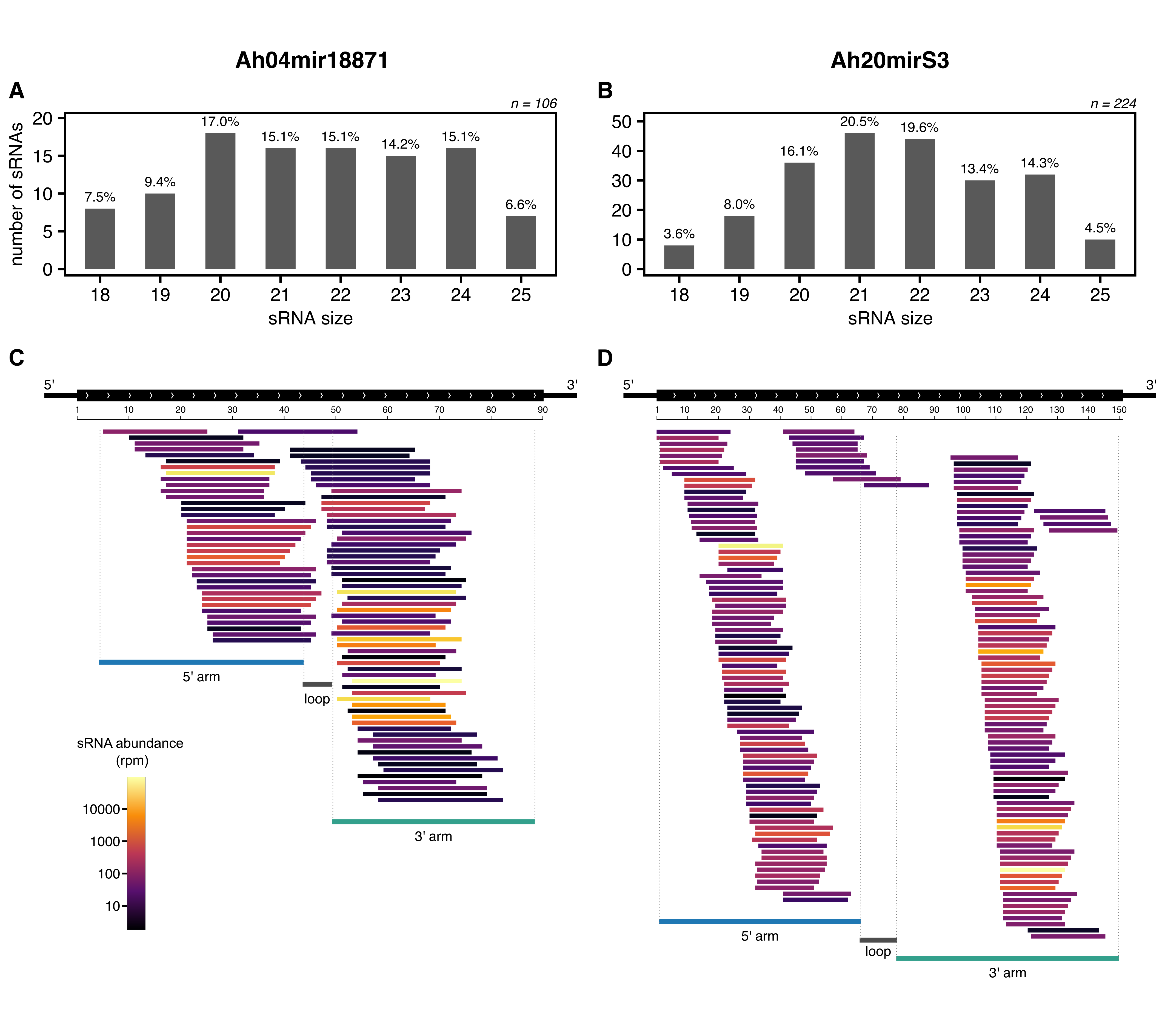

Supplement: S8 Fig — Size distribution of sRNAs derived from Ah04mir1887 (A) and Ah20mirS3 (B). (C-D) Genomic representation of the Ah04mir1887 and Ah20mirS3 loci, and their respective sRNAs. sRNAs are colored according to their abundance level. (TIFF) [file pgen.1012127.s014.tiff]

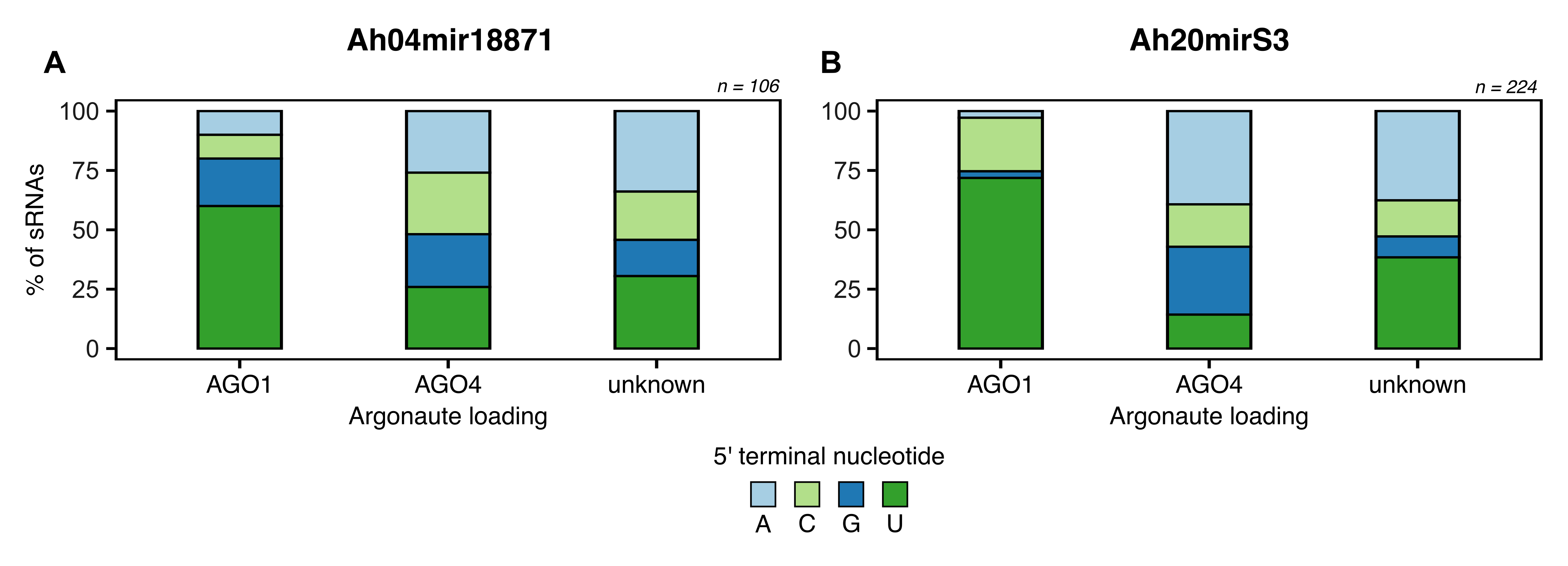

Supplement: S10 Fig — (TIFF) [file pgen.1012127.s016.tiff]
