# Supplementary material for: Dominance modifiers at the Arabidopsis self-incompatibility locus retain proto-miRNA features and act through non-canonical pathways
Source: PLoS Genet. 2026 Apr 20;22(4):e1012127. doi: 10.1371/journal.pgen.1012127 (PMC13128112; doi:10.1371/journal.pgen.1012127)
Supplement: Attachment — Submitted filename: batista-review.pdf [file pgen.1012127.s019.pdf]

Summary

Batista *et al.* investigate the molecular mechanism causing S-allele dominance interactions using a reverse genetics approach in Arabidopsis. The present manuscript builds on previous work demonstrating that sRNA precursors (similar to miRNAs) underly dominance interactions between *S*-alleles in heterozygous plants.

The authors report two key findings: that recessive allele expression is reduced through non-canonical transcriptional/non-transcriptional silencing pathways and that features of sRNA processing enable flexible targeting of a large number of recessive alleles. These findings will be of interest to a broad readership, revealing molecular details of a specific self-incompatibility mechanism with broader implications for network function. Pending revisions, I believe this work will be suitable for publication at *PLOS* Genetics. In addition to several minor comments detailed below, I have two main concerns related to 1. the motivational framing in the introduction and 2. the overall lack of statistical details.

Major Comments

1. The finding that sRNA precursors do not act through canonical pathways is important for establishing future experimental directions (which are thoughtfully outlined in the Discussion). However, given that this is in many ways a null result, it is important to clearly outline why one might expect that sRNA precursors act through the RdDM pathway in the Introduction. As it stands, I do not find L50–57 sufficient motivation. Some of the reasoning outlined in L128–138 should be included in the Introduction to increase the overall readability and impact of the study.

2. Although I find the data presented convincing, it is difficult to truly assess given the current lack of statistical details throughout the manuscript. For example, in Figure 1C, the variance for number of pollen grains germinated in the pollV appears high making visual assessment of difference between treatments problematic. Additionally, the legend accompanying Figure 1 S4 describes "the predicted model" but I can't find any mention of what that model is.

Minor Comments

- L22: Defining the *S*-locus as the "self-incompatibility locus" is misleading given that many interactors in the self-incompatibility pathway are found elsewhere in the genome.

- L31: I find this explanation a bit confusing given that dominance interactions do not equally maximize reproductive success for all alleles.

- L93, Figure 1, and throughout: Use of "female" and "male" parents is confusing and potentially misleading as it suggests unique individuals. One possibility is to change to "pistil" and "pollen" parent which is more biologically accurate and specific.

- L121: Was this difference significant? What statistical test was used?

- L161: My understanding is that the PTGS pathway was not tested in this study, and this assertion is therefore a bit of an overstatement. However, I do agree that the features outlined in L127–133 are inconsistent with PTGS.

- L178: The language "no clear predominance" is unclear—what evidence is this claim based on beyond looking at the distribution?

- L258: Typo for Pol II?

- L290: The introduction states that the hypothesis of Smi and Smi2 using RdDM pathway in Brassica has never been empirically tested so it's a bit unclear to me how the results contrast with previous studies (given that methylation was not measured here).

- L331: *S*-RNase based self-incompatibility has been observed in many plant families in addition to Solanaceae

- L331: I agree that superficially this mechanism resembles that of collaborative non-self recognition by F-box genes within the *S*-RNase S-locus. However, it is not clear to me how drawing this parallel is useful to the author's purpose here. Are there features of self-recognition (or self-incompatibility *per se*?) pathways that make generalism more likely? This point again becomes critical for the Discussion section beginning L342.

- L350: While this hypothesis is compelling, the "specific selective constraints" discussed need to be outlined more clearly.

- L438: Was the total number of pollen grains deposited in each pollination quantified in addition to the number germinated? There appears to be quite a large spread even within treatments. Representative images of pollinated pistils (stained with aniline blue) would be useful.

- L455: Were these phylogenetic relationships inferred in the present study?

- L468: How were buds sampled standardized to be developmentally immature? Relative to anther dehiscence or some other developmental stage?

Figures

- Figure 1C: The number of germinated pollen grains appears to saturate at 30. Is this true or an artifact of the presentation? If this is true biologically, why?

- Figure 1C/D: The statistical methods used to analyze these data is missing. Are the differences shown statistically significant? How were repeated measurements made on the same individual

(across the five biological replicates) handled? Also, the use of the same five colors across the separate treatments (e.g., with and without Ah04mir1887 in panel C) suggests paired measurements. From reading the methods, my understanding is that this is not the case. Also, the legend should include what the "x" in the figure represents.

- Figure 1 S4: This is an interesting finding and a clever analysis. However, more details on the statistical model used are necessary. For example, how was non-independence between multiple measurements made on the same individual plant measured?

- Figure 4: Formatting for the "*S*-locus" text should be consistent with the rest of the manuscript

- Figure 4 legend: More detail is needed to sufficiently understand this figure. Specifically, it is not clear what is plotted in the summary plots for each feature.
