# Supplementary material for: Dominance modifiers at the Arabidopsis self-incompatibility locus retain proto-miRNA features and act through non-canonical pathways
Source: PLoS Genet. 2026 Apr 20;22(4):e1012127. doi: 10.1371/journal.pgen.1012127 (PMC13128112; doi:10.1371/journal.pgen.1012127)
Supplement: Attachment — Submitted filename: response to reviewers.pdf [file pgen.1012127.s021.pdf]

Dear Editors,

We would like to thank you and the three reviewers for the positive and insightful evaluation of our manuscript. We were encouraged to read that our work represents a “*very interesting study that makes an important contribution*” (Reviewer #1), that it “*presents a clever design and a powerful genetic system [...]. Most of the conclusions are well supported, and the discussion is very appropriate*.” (Reviewer #2) and that “*These findings will be of interest to a broad readership, revealing molecular details [...] with broader implications for network function*” (Reviewer #3).

The main comment, which was raised by all three reviewers, was the absence of formal statistical tests supporting our claim that transcript levels and the strength of the self-incompatibility response vary between lines. In response to this, we have now implemented appropriate statistical tests throughout the manuscript. These analyses fully support our original interpretation that the sRNA precursors strongly silence their *SCR01* targets, including in mutant backgrounds deficient in the RdDM pathway. We thank the reviewers for flagging this shortcoming, and we feel that our main conclusions now hold even more firmly.

The first two reviewers also requested direct measurements of DNA methylation. We had initially shied away from this because the small size of Arabidopsis flowers makes it difficult to isolate the anther tapetum (the tissue of interest) so whole buds must be used, where the tapetum is only a small and unknown fraction of cells. Nevertheless, triggered by the reviewers’ request, we performed Bisulfite Amplicon Sequencing (BSAS) on young floral buds and found no evidence that sRNA-mediated repression of *SCR01* is associated with increased DNA methylation at sRNA target sites. This aligns with the lack of RdDM pathway involvement shown with the phenotypic data. However, as detailed below and in the manuscript, interpreting these results is complicated by the high baseline methylation at the target sequences in these bulk tissues.

We have carefully addressed these and all additional comments and believe that the revised manuscript is substantially improved and strengthened as a result.

A detailed point-by-point response is provided below.

Reviewers' comments:

<u>Reviewer #1</u>

This study investigates the biogenesis and mode of action of small RNAs that act as dominance modifiers in the hierarchy of S-locus alleles of Arabis. To do so, the authors have reconstituted the A. halleri self-incompatibility response in Arabidopsis thaliana by expressing a cognate pair of SRK and SCR alleles, with or without small-RNA loci that are thought to target and suppress expression of the particular SCR allele used. By combining this system with mutations in the canonical RdDM pathway, the authors show that the sRNA-mediated reduction in SCR transcript levels is independent of the RdDM pathway. They go on to perform sRNA sequencing on appropriate genotypes and find that the sRNA precursors are processed into a multitude of sRNAs of different length and sequence; are loaded into AGO1 or AGO4 and probably also into other unknown AGOs; and can target a large number of SCR alleles in A. halleri. Often sRNAs made from the same precursor target several SCR alleles, and many SCR allele is targeted by multiple different sRNAs. Based on this, the authors propose that the sRNA loci have retained features of proto-miRNAs that are processed into many different sRNA species and can trigger different effector pathways to silence SCR expression from recessive S-locus haplotypes.
This is a very interesting study that makes an important contribution to our understanding of the dominance hierarchy between S-locus alleles in the Brassicaceae and suggests a different mode of action of the sRNA modifiers compared to Brassica.
The following comments should be addressed to further strengthen the manuscript.

1. Figure 1: The difference between lines with and without the sRNA construct is pretty clear, but for completeness' sake it would be good to add the result of statistical tests.

As requested by all reviewers, we have now included statistical analyses for both the SI phenotypic response and changes in *SCR01* expression. The statistical tests, including details on the number of replicates, test type, and correction for multiple comparisons, are described in the Methods section and in the corresponding figure legends. Overall, these analyses fully support our original conclusions and do not alter any of the main findings or their interpretation.

2. Predicted targeting of SCR01 promotor (lines 106-107): This is not entirely clear from Figure 1 S1, possibly due to the smoothing of the curves using a 20 nt sliding window. Are the authors certain that the miR target site is not in the 5' UTR?

For improved clarity, we have updated Figure 1 S1 to show the aligned reads directly and relative to the *SCR01* translation start site (ATG). This analysis confirms that the Ah04mir1887 target site is located approximately 5 bp upstream of the main transcription start site of *SCR01*, which itself is positioned around -30 to -10 bp from the translation start site. These data indicate that the Ah04mir1887 target site is not included in the 5'UTR of the predominant *SCR01* transcript isoform.

3. Lines 113-116: Why did the others not make a new sRNA expressing line that only expresses Ah20mirS3? Can they please comment on this?

To isolate the effect of Ah20mirS3, we chose to perform site-directed mutagenesis of the Ah20mir1887 target site in *SCR01* rather than generate a new transgenic line expressing Ah20mirS3 alone. This strategy was adopted because of the very close physical proximity between the two precursors, as well as to preserve the native genomic context surrounding the Ah20mirS3 precursor, which may contain regulatory elements required for its correct expression and processing. As the nature and precise location of these regulatory sequences are unknown, we considered modifying the target site was a more conservative and controlled approach than altering the native sRNA precursor locus itself. We have added this clarification to the Methods section.

4. Lines 149-152 "And while…(Fig. 1D).": This sentence is rather unclear and seems to give the wrong impression. Please rephrase.

Thank you for pointing out this unprecise formulation. We have corrected this sentence, which should hopefully now be clearer: "*And while in the ago4 ago6 mutant background the reduction in SCR01 transcript led to a compatible pollen phenotype, in both polIV and polV mutant backgrounds the reduction in SCR01 transcript level was significant but insufficient to elicit a compatible pollen phenotype (Fig. 1D) .*"

5. Lines 152-158: Statistical tests would be good to show that the level of SCR01 expression is indeed higher in polIV and polV mutants without the sRNA than in wt without sRNA. Also, do the authors have another transgenic line for the SCR01*mir1887 construct that perhaps escapes the suggested transgene silencing? This would be useful to strengthen the authors' interpretation versus the alternative that the sRNA-mediated silencing is less efficient in the polIV and polV backgrounds.

Unfortunately, we do not have additional independent *SCR01*mir1887* transgenic lines in the RdDM mutant backgrounds. Early in this study, and in an effort to minimize the number of possible confounding factors, we chose to establish a single stable representative reference line for *SCR01*mir1887* and use it for backcrosses to the sRNA precursor and all RdDM mutant lines. This strategy was chosen because generating multiple independent transgenic lines for every combination of *SRK01*, *SCR01*, and sRNA precursor in each RdDM mutant background would be unfeasible time-wise, and using a single reference line allows for consistent comparisons of phenotypic and expression data across all genetic backgrounds. We have clarified this in the Methods section.

We have now performed formal statistical comparisons of *SCR01* transcript levels in the presence and absence of sRNA precursor in the RdDM mutant backgrounds versus the wild-type background (Fig. 1C and D, Table S2 and S3). Indeed these tests show that *SCR01* is significantly highly expressed in the *polIV* and *polV* backgrounds when compared to the wild-type control (both in the presence and absence of Ah20mirS3). The newly added statistical tests also show that Ah20mirS3 has a significant effect in reducing *SCR01* expression in all mutant backgrounds, supporting our conclusion that this precursor is still functional and able to reduce *SCR01* expression in these mutant backgrounds.

6. Line 176: Should this not be mir1887?

Yes, thank you for pointing out this typo.

7. Figure 3: It is not clear to me why mir1887 is not considered in the dominance interactions by the Ah20 allele. Can the authors please explain this? Should small RNAs derived from the mir1887 locus not also be made in plants heterozygous for Ah20?

The reason for focusing on mirS3 rather than mir1887 in this context is that the latter is not expected to be involved in the dominance of Ah20 over Ah01. Ah20mir1887 is indeed expressed (and as such represented in the row “other mirs”), but it is predicted to be involved in the regulation of Ah20 over class II alleles (Ah03, Ah33 and Ah19). We have clarified the legend of Figure 3 to reflect this.

8. Lines 286-289: Is there any chance of repeating the experiment in an ago1 ago4 ago6 triple mutant background?

We generated *ago4 ago6* loss-of-function mutants in the C24 background to test the hypothesis that RdDM would be required for sRNA precursor function. Given the results derived from this experiment, we completely agree that evaluating this phenotype in an *ago1* background would be of significant interest. However, *ago1* null mutants are generally sterile (Vaucheret et al. 2004, Genes & Development), making a *ago1 ago4 ago6* triple mutant background unsuitable for this experimental setup. In principle, hypomorphic *ago1* alleles could be used, but none are available in the C24 background, which is required to reconstitute the SI reaction (see Methods section *“Generation and validation of CRISPR/Cas9 mutants”*). Because of this, generating the necessary lines to test the role of AGO1 requires substantial time and effort, placing this experiment beyond the scope of the current study. Nevertheless, this work is ongoing in our lab as a follow-up to the findings reported here.

9. Lines 342 onwards: What is known about the evolutionary age of the sRNA loci mediating the dominance hierachy? In other words, for how long have they maintained their proto-miRNA features? Can closely related sRNA loci like mir1887 and mirS3 also be found at the genome-sequence level for example in C. grandiflora? If yes, this would strengthen the argument that their proto-miRNA features are functionally relevant. If not, the alternative would be that the ones in A. halleri are simply young and haven't been streamlined by evolution yet to produce single, well-defined sRNAs.

This is a very good point. Indeed, our initial analysis (Durand et al. 2014, Science) included a phylogenetic reconstruction that showed that the different sRNA loci have drastically different evolutionary “ages”. Specifically, while it is clear that some sRNA loci have emerged recently (*e.g.* mirS4, mir867), the sRNA loci included in the present study (mirS3 and mir1887) are shared among *S*-alleles that are extremely diverged, and are also shared across distantly related Brassicaceae species. Hence, their emergence can be traced back to the diversification of the whole family, about 30 My ago. Overall, it is thus clear that mirS3 and mir1887 have maintained their proto-miRNA features for very long evolutionary times. We have rephrased this part to drive this point home more prominently: “*Our previous work has shown that S-locus sRNA precursors vary widely in evolutionary age: some, such as mirS4 and mir867, have emerged relatively recently, while others trace back to the diversification of the Brassicaceae family approximately 30 Mya (19). It is therefore clear that these older sRNA precursors, including mirS3 and mir1887 studied here, have retained proto-miRNA features over long evolutionary periods.”*

10. It is a real pity that DNA methylation of the transgenes has not been directly tested. I can see the authors' argument that this should only be expected in the tapetum, and so should be difficult to study in bulk samples. I also appreciate that setting up FACS or INTACT to isolate tapetum nuclei is beyond the scope of the present study. However, doing bisulfite sequencing from young anthers at a high coverage should be able to detect methylated DNA from the subpopulation of tapetum cells. This seems quite feasible and doing so would greatly strengthen the manuscript.

We agree that directly assessing DNA methylation at the *SCR01* transgene would strengthen the study. We therefore performed Bisulfite Amplicon Sequencing (BSAS) on young floral buds to test whether sRNA-mediated repression of *SCR01* is associated with DNA methylation changes at the target sites (Figures 1 S5 and 1 S6 and Tables S4 and S5). We used this approach in order to achieve high sequencing depth (~1400x per cytosine), with the goal of detecting methylation changes arising from the subpopulation of tapetum cells, even in bulk tissue samples. At first glance, our results reveal no significant differences in DNA methylation in the presence of Ah04mir1887 or Ah20mirS3. Taken at face value, this seems to indicate that the reduction in *SCR01* transcript abundance is not associated with changes in DNA methylation, and would *a priori* be consistent with the lack of phenotypic effects we observed in RdDM mutant backgrounds (Figure 1).

However, we caution against a too direct interpretation of these results for several reasons. First, the proportion of tapetal cells in our bulk samples is unknown, and despite controlling for the developmental stage of the collected tissue, we cannot exclude variation of this proportion among samples. Second, our analysis revealed an unusually high baseline DNA methylation level at *SCR01* target sites (75-95% methylated cytosines across the three sequence contexts, even in the absence of the sRNA precursor locus), similar to levels typically observed at transposable elements rather than genic regions. Understanding the reason for such high TE-like levels of DNA methylation (biological reality or technical artifact) will require more efforts, and for now this leads us to doubt the relevance of the conclusions we can draw from the BSAS experiment described above. Instead, we have started to implement a FACS cell sorting protocol to isolate nuclei and directly measure DNA methylation from tapetal cells. This new experiment is technically ambitious and will pose its own challenges, and will be part of a follow-up study within the framework of an ongoing PhD project. We have included the BSAS data along with these caveats in the revised manuscript, and we highlight both the interest and feasibility of future experiments to directly assess tapetum-specific DNA methylation in the Results and Discussion sections.

Reviewer #2

Batista et al. report a molecular dissection of the sRNA-based self-incompatibility (SI) dominance across A. halleri alleles. To this end, the authors recreated the self-incompatibility reaction in the model plant A. thaliana by introducing different A. halleri alleles for the SRK01 receptor and its cognate SCR01 ligand in the C24 accession. They then introduced distinct dominant A. halleri sRNA precursor loci to test their molecular pathways as well as the incompatibility reactions. The authors further tested this system in mutant backgrounds for distinct components of canonical RNA-dependent DNA methylation pathways and found that these mutants do not alter the incompatibility reactions, excluding a role for canonical RdDM. This is an important result. The authors performed additional analyses to characterize the repertoire and AGO loading of siRNAs produced by the sRNA precursor loci, and conclude that they operate through an unconventional post-transcriptional gene silencing pathway, triggered by miRNA-like precursors.

Overall, the manuscript presents a clever design and a powerful genetic system that will be important for researchers studying the genetic basis of self-incompatibility. Most of the conclusions are well supported, and the discussion is very appropriate.

I have nonetheless identified some issues that the authors should revise.

Fig. 1C and Fig. 1D. The plots currently show differences, but the results are lacking statistical testing. Please include which test was used, what the n represents, and how multiple comparisons were handled. Importantly, it is unclear whether siliques are treated as independent experimental units or pseudo-replicates, and how many independent transgenic lines were used for each experiment.

As requested by all reviewers, we have now included statistical analyses for both the SI phenotypic response and changes in *SCR01* expression. The statistical tests, including details on the number of replicates, test type, and correction for multiple comparisons, are described in the Methods section and in the corresponding figure legends. Overall, these analyses fully support our original conclusions and do not alter any of the main findings or their interpretation.

For both assays, three to four biological replicates were used. Each biological replicate corresponds to an individual plant that served as the pollen donor in the SI phenotypic assay and as the source of RNA for the *SCR01* expression assay; the same plant is represented by the same colored dot across both plots. In the SI assay, each pollen donor was used to pollinate multiple SRK01-expressing pistils, which serve as technical replicates and are indicated by the same color within this plot.

Regarding transgenic lines, multiple independent single-insertion lines were initially generated and phenotyped for *SRK01*, *SCR01* and *SCR01*mir1887*, and the sRNA precursors Ah20mirS3 and Ah04mir1884, and consistent phenotypes were observed across lines (see Methods). Because this study required multiple combinations of *SRK01*, *SCR01*, and sRNA precursor transgenes in both wild-type and RdDM mutant backgrounds, we selected a single representative reference line for each transgene to use in subsequent crosses. This approach ensured consistent comparisons across genetic backgrounds and was necessary because generating multiple independent lines for every combination would be unfeasible. We have clarified this in the Methods section.

Fig. 1D (polIV/polV backgrounds). Introduction of Ah20mirS3 in polIV and polV backgrounds still appears to yield an incompatible pollen phenotype (low pollen germination). This is unexpected if the interpretation is that the effect is independent of canonical RdDM. Please clarify the reason for this outcome (e.g., elevated basal SCR01 transcript levels in these mutants and incomplete reduction below the phenotypic threshold), and ensure that the text and figure are fully consistent.

We agree with the reviewer's interpretation: the persistence of an incompatible pollen phenotype in the *polIV* and *polV* backgrounds upon introduction of Ah20mirS3 is best explained by the elevated basal levels of *SCR01* transcript in these mutants.

We have now performed statistical analyses comparing *SCR01* expression with and without Ah20mirS3 in RdDM mutant backgrounds versus wild type (Fig. 1C and D, Table S3). These analyses confirm that *SCR01* expression is significantly higher in *polIV* and *polV* mutants than in wild type, both

in the absence and in the presence of Ah20mirS3. Importantly, Ah20mirS3 still causes a significant reduction in *SCR01* expression in all backgrounds, indicating that the sRNA precursor remains functional in these mutants. However, in the RdDM mutants this reduction is insufficient to lower *SCR01* expression below the threshold required to confer pollen compatibility, resulting in the observed incompatible phenotype. We have revised the text and figure legend to reflect this interpretation.

sRNA pathway benchmarks. The authors reports atypical size/processing features for dominance modifier sRNAs, but this claim is lacking controls, For instance, does the atypical siRNAs profile is biological, or an artifact of the library? The Authors should include analyses of siRNAs size and 5′ nucleotide composition for representative canonical RdDM (24-nt heterochromatic siRNAs), PTGS/RDR6-dependent siRNAs (e.g., ta-siRNAs), and annotated miRNAs, alongside the S-locus-derived sRNAs. If AGO-IP is used to infer loading, it would also help to show expected enrichment of these "canonical targets" in AGO4 vs AGO1 as positive controls for the IPs.

We thank the reviewer for this important suggestion. To address this concern, we now have included a benchmarking analysis of canonical sRNA populations in our *Arabidopsis thaliana* libraries (Figure 2 S5). We examined sRNA size distribution, 5′ terminal nucleotide composition, and AGO association across well-characterized classes, including canonical miRNAs expressed in floral buds (miR159, miR162, miR165/166, miR167, miR160), RdDM loci (Pol IV/Pol V-dependent Group E loci from Blevins et al., 2015), TAS loci (TAS1a/b), and all annotated TEs. These controls display the expected features: miRNAs and tasiRNAs are predominantly 21 nt, enriched in AGO1, and show a 5′ U bias, whereas RdDM- and TE-derived siRNAs are enriched for 24 nt species with a 5′ A bias and preferentially associate with AGO4. These results confirm that our libraries and AGO immunoprecipitations accurately capture canonical sRNA populations, supporting their quality. Therefore, the atypical features observed for the dominance modifier sRNAs are unlikely to reflect technical artifacts and instead represent genuine biological differences.

The authors interpret the results as being mediated by an unconventional PTGS pathway. However, I wonder whether miRNA-like trigger followed by transitivity (secondary siRNA production and amplification on the SCR01 transcript) could also fit. Please provide additional analyses to assess whether SCR01 shows signatures of transitivity upon expression of the dominance modifiers, including (i) mapping and coverage plots of sRNAs along the SCR01 transcript and flanking regions in the presence vs absence of Ah20mirS3/Ah04mir1887, (ii) size-class and strand-bias profiles of SCR01-mapping sRNAs (in particular, whether new 21–22 nt species appear and spread away from the predicted target site), and (iii) if feasible, an analysis of phasing or positional enrichment relative to the predicted target site. These analyses would help clarify whether amplification via secondary siRNAs could contribute to the observed SCR01 downregulation.

We agree that a process involving a miRNA-like trigger followed by transitivity could in principle have been envisioned. Although we do not have sequencing experiments where we can directly compare the presence/absence of sRNA precursors in *A. thaliana*, throughout the years we sequenced multiple *A. halleri* genotypes with a wide range of *S*-allele combinations, and never observed considerable sRNA accumulation at *SCR* loci, except for a few selected islands which largely correspond to TE regions. We therefore exclude the possibility that secondary sRNAs and transitivity would be triggered. To illustrate this, we have provided exemplifying genome browser snapshots for two *SCR* alleles (*SCR01* and *SCR04*) in Fig. 2 S4, and formally mention this possibility in the Discussion session.

"PTGS unlikely because the target is promoter/intron". The argument that PTGS is unlikely because PTGS substrates are "generally thought to be the mature mRNA" is not fully convincing on its own, since nuclear/transcript-associated pathways could still operate. Indeed, the manuscript later invokes non-canonical pathways…. Please qualified the "PTGS unlikely" claim and explicitly discuss alternative RNA-based scenarios that remain compatible with the data, for example promoter-proximal transcripts and transitivity.

We thank the reviewer for these suggestions. In line with the previous reply, we have never found empirical evidence for any of the suggested scenarios, such as transitivity, antisense transcripts or promoter-proximal transcripts in any of our sequencing experiments. We have now explicitly mentioned this and referred to these scenarios in several instances of the Discussion, to provide the reader with a broader view.

Many conclusions in the manuscript rely on a hard cutoff (score ≥ 18) to define “functional” sRNAs and build the interaction network. It would be interesting to provide as sensitivity test to show whether the conclusions are robust to changes in the threshold (e.g., 16, 17, 18, 19, 20...) and clarify how mismatches and G:U pairs are weighted in the scoring (even if detailed in the prior reference, a short reminder in the methods section would help).

The choice of a score ≥ 18 to define functional sRNA-target interactions is based on our previous work, in which the relationship between *SCR* expression and sRNA-target base-pairing score was carefully evaluated across a wide range of alignment scores (Burghgraeve et al. 2019, Genetics). In that study, *SCR* repression was associated with a simple threshold model, with interactions scoring ≥18 consistently linked to transcriptional silencing (Fig. 4 in Burghgraeve et al. 2019, Genetics). Notably, the transition between high and low *SCR* expression was abrupt around this value, arguing against a gradual quantitative model and supporting the use of a hard cutoff.

Because the conclusions in the present manuscript rely on the same interaction logic and involve the same molecular players, we used this experimentally validated threshold rather than re-optimizing it. We now explicitly refer to this prior sensitivity analysis in the manuscript and clarify the scoring scheme in the Methods section. Briefly, sRNA targets were predicted using the Smith–Waterman algorithm with matches scored +1, mismatches −1, gaps −2, and G:U wobbles −0.5.

It would be interesting to test DNA methylation of interactions A and B to rule out unconventional RdDM-directed TGS.

(See also question 10 from Reviewer #1. The same response follows below.)

We agree that directly assessing DNA methylation at the *SCR01* transgene would strengthen the study. We therefore performed Bisulfite Amplicon Sequencing (BSAS) on young floral buds to test whether sRNA-mediated repression of *SCR01* is associated with DNA methylation changes at the target sites (Figures 1 S5 and 1 S6 and Tables S4 and S5). We used this approach in order to achieve high sequencing depth (~1400x per cytosine), with the goal of detecting methylation changes arising from the subpopulation of tapetum cells, even in bulk tissue samples. At first glance, our results reveal no significant differences in DNA methylation in the presence of Ah04mir1887 or Ah20mirS3. Taken at face value, this seems to indicate that the reduction in *SCR01* transcript abundance is not associated with changes in DNA methylation, and would *a priori* be consistent with the lack of phenotypic effects we observed in RdDM mutant backgrounds (Figure 1).

However, we caution against a too direct interpretation of these results for several reasons. First, the proportion of tapetal cells in our bulk samples is unknown, and despite controlling for the developmental stage of the collected tissue, we cannot exclude variation of this proportion among samples. Second, our analysis revealed an unusually high baseline DNA methylation level at *SCR01* target sites (75-95% methylated cytosines across the three sequence contexts, even in the absence of the sRNA precursor locus), similar to levels typically observed at transposable elements rather than genic regions. Understanding the reason for such high TE-like levels of DNA methylation (biological reality or technical artifact) will require more efforts, and for now this leads us to doubt the relevance of the conclusions we can draw from the BSAS experiment described above. Instead, we have started to implement a FACS cell sorting protocol to isolate nuclei and directly measure DNA methylation from tapetal cells. This new experiment is technically ambitious and will pose its own challenges, and will be part of a follow-up study within the framework of an ongoing PhD project. We have included the BSAS data along with these caveats in the revised manuscript, and we highlight both the interest and feasibility of future experiments to directly assess tapetum-specific DNA methylation in the Results and Discussion sections.

Reviewer #3:

Batista *et al.* investigate the molecular mechanism causing S-allele dominance interactions us- ing a reverse genetics approach in Arabidopsis. The present manuscript builds on previous work demonstrating that sRNA precursors (similar to miRNAs) underly dominance interactions between *S*-alleles in heterozygous plants.

The authors report two key findings: that recessive allele expression is reduced through non-canonical transcriptional/non-transcriptional silencing pathways and that features of sRNA processing enable flexible targeting of a large number of recessive alleles. These findings will be of interest to a broad readership, revealing molecular details of a specific self-incompatibility mechanism with broader implications for network function. Pending revisions, I believe this work will be suitable for publication at *PLOS* Genetics. In addition to several minor comments detailed below, I have two main concerns related to 1. the motivational framing in the introduction and 2. the overall lack of statistical details.

Major Comments

1. The finding that sRNA precursors do not act through canonical pathways is important for establishing future experimental directions (which are thoughtfully outlined in the Discussion). However, given that this is in many ways a null result, it is important to clearly outline why one might expect that sRNA precursors act through the RdDM pathway in the Introduction. As it stands, I do not find L50–57 sufficient motivation. Some of the reasoning outlined in L128–138 should be included in the Introduction to increase the overall readability and impact of the study.

   We thank the reviewer for this suggestion and we have modified the Introduction text in several places, adding a more detailed background as to why RdDM was tested in this study.

2. Although I find the data presented convincing, it is difficult to truly assess given the current lack of statistical details throughout the manuscript. For example, in Figure 1C, the variance for number of pollen grains germinated in the polIV appears high making visual assessment of difference between treatments problematic. Additionally, the legend accompanying Figure 1 S4 describes “the predicted model” but I can’t find any mention of what that model is.

   As requested by all reviewers, we have now included statistical analyses for both the SI phenotypic response and changes in *SCR01* expression. The statistical tests, including details on the number of replicates, test type, and correction for multiple comparisons, are described in the Methods section and in the corresponding figure legends. Overall, these analyses fully support our original conclusions and do not alter any of the main findings or their interpretation. We have also provided more details in the legend of Figure 1S4 regarding the described model.

Minor Comments

- L22: Defining the *S*-locus as the “self-incompatibility locus” is misleading given that many interactors in the self-incompatibility pathway are found elsewhere in the genome.

  This terminology is deeply rooted in the field, and is a very widespread and consensus nomenclature for this locus (see: https://doi.org/10.1105/tpc.13.3.627), where the genes were originally cloned in 2001 and can even be traced back at least as early as the foundational classical genetics works by Emmerson in 1939 (10.1093/genetics/23.2.190). We have nevertheless added a clarification in the Introduction that additional signalling processes essential for the self-incompatibility pathway are mediated by genes outside the S-locus.

- L31: I find this explanation a bit confusing given that dominance interactions do not equally maximize reproductive success for all alleles.

  We agree that dominance interactions do not equally maximize reproductive success for all alleles in a population-level sense. Rather, dominance maximizes reproductive success at the level of individual genotypes. In a diploid individual, expression of only one *SCR* allele ensures that pollen is rejected only by females carrying that specific allelic specificity. In contrast, simultaneous expression of two different *SCR* alleles would cause rejection by

females carrying either specificity, thereby reducing the number of compatible mates. We have revised the sentence to clarify this point.

- L93, Figure 1, and throughout: Use of “female” and “male” parents is confusing and potentially misleading as it suggests unique individuals. One possibility is to change to “pistil” and “pollen” parent which is more biologically accurate and specific

  We have adjusted the text accordingly throughout the manuscript.

- L121: Was this difference significant? What statistical test was used?

  Yes, this difference was significant for both comparisons. Please refer to the answer to Major Comment 2 for further information about the addition of statistical tests.

- L161: My understanding is that the PTGS pathway was not tested in this study, and this assertion is therefore a bit of an overstatement. However, I do agree that the features outlined in L127–133 are inconsistent with PTGS.

  We have toned down the claims made in this Results section and added additional hypotheses for the mode of action of sRNA precursors at several different locations in the Discussion section.

- L178: The language “no clear predominance” is unclear—what evidence is this claim based on beyond looking at the distribution?

  We have clarified this sentence to reflect that there are no overrepresented size categories, based on the observed size distribution.

- L258: Typo for Pol II?

  Indeed, thanks for pointing this out, it has been corrected.

- L290: The introduction states that the hypothesis of Smi and Smi2 using RdDM pathway in Brassica has never been empirically tested so it’s a bit unclear to me how the results contrast with previous studies (given that methylation was not measured here).

  We meant to indicate that our results contrast with the previously published hypothesis in *Brassica* that *de novo* deposition of DNA methylation underlies *Smi* and *Smi2* action, which was based on methylation measurements in that study (Shiba et al. 2005, Nature Genetics). We have now performed direct methylation measurements ourselves and the manuscript has been revised accordingly in multiple sections to reflect these new data. Our phenotypic data, together with the newly added DNA methylation measurements, suggest sRNA precursor action in *Arabidopsis* is not dependent on DNA methylation, contrasting with the hypothesis proposed in *Brassica.* We guide the reviewer to answer 10 to Reviewer 1 (page 4) for more details and caveats on the newly added DNA methylation measurements.

- L331: *S*-RNase based self-incompatibility has been observed in many plant families in addition to Solanaceae

  We have modified the text and provided references accordingly.

- L331: I agree that superficially this mechanism resembles that of collaborative non-self recognition by F-box genes within the *S*-RNase S-locus. However, it is not clear to me how drawing this parallel is useful to the author’s purpose here. Are there features of self-recognition (or self- incompatibility *per se*?) pathways that make generalism more likely? This point again becomes critical for the Discussion section beginning L342.

  The reference to Solanaceae was intended as a conceptual parallel: both systems achieve generalism in their self-incompatibility networks, but via distinct molecular mechanisms. In Solanaceae, generalism arises from SLF protein interactions with multiple S-RNases,

whereas in *A. halleri*, generalism relies on the molecular properties of sRNA precursors producing multiple sRNAs with distinct targeting spectra. We have revised the Discussion to clarify this parallel.

- L350: While this hypothesis is compelling, the "specific selective constraints" discussed need to be outlined more clearly.

  Thank you for pointing out this unclear sentence. We now make this argument more explicit as: "*We hypothesize that natural selection promotes diversification of the targeting spectrum of these S-locus sRNA precursors towards a broader set of recessive S-alleles, rather than towards highly specific interactions as seen in canonical miRNAs (56). This selective pressure likely contributed to the persistence of proto-miRNA features over extended evolutionary timescales, preventing their maturation into canonical miRNAs, which would likely compromise their ability to target multiple S-alleles, and thus the structure of the dominance network.*"

- L438: Was the total number of pollen grains deposited in each pollination quantified in addition to the number germinated? There appears to be quite a large spread even within treatments. Representative images of pollinated pistils (stained with aniline blue) would be useful.
- Figure 1C: The number of germinated pollen grains appears to saturate at 30. Is this true or an artifact of the presentation? If this is true biologically, why?

  We used flowers with dehiscent anthers for pollination, and applied pollen in excess to achieve saturation of the pistil (on the order of several hundreds of grains, compared to 40–60 ovules per pistil). The observed variation therefore reflects differences in pollen germination success, rather than insufficient pollen deposition. Because hundreds of pollen grains are retained on the stigma in close proximity, it is technically challenging to accurately count each individual deposited/germinating grain. For this reason, we previously established that counting 30 or more germinated pollen grains reliably reflects a robust compatible reaction (Durand et al. 2014, Science), and we cap counts at 30 in the analysis. To illustrate these points, representative aniline blue-stained pistils are now provided in Fig. 1 S7. We have also further detailed these aspects in the Methods section.

- L455: Were these phylogenetic relationships inferred in the present study?

  Yes, we have modified the text to reflect this.

- L468: How were buds sampled standardized to be developmentally immature? Relative to anther dehiscence or some other developmental stage?

  Relative to anthesis and following Smyth's flower development stages (Smyth et al. 1990, Plant Cell). Detailed information of the bud stages is now included for AGO IP experiments, as well as qPCR experiments.

Figures

- Figure 1C/D: The statistical methods used to analyze these data is missing. Are the differences shown statistically significant?

  Please refer to the answer to Major Comment 2.

- How were repeated measurements made on the same individual (across the five biological replicates) handled? Also, the use of the same five colors across the separate treatments (e.g., with and without Ah04mir1887 in panel C) suggests paired measurements. From reading the methods, my understanding is that this is not the case. Also, the legend should include what the "x" in the figure represents.

  In the SI phenotype panels, the same-colored dots represent technical replicates, i.e.: multiple pistils from a single SRK01 individual pollinated with the same pollen donor. Each biological

replicate corresponds to a distinct pistil/pollen donor combination. The assay is paired at the phenotype-qPCR level, meaning that the same plant used as pollen donor in the phenotypic assay (top panel) is represented by the same color in the corresponding *SCR01* expression panel (bottom). This pairing allows a direct comparison between *SCR01* transcript levels and the measured pollen germination phenotype. These details have been clarified in both the figure legend and the Methods section.
Additionally, the “x” in the figure indicates the pollen deposition of the indicated male genotype on SRK01-expressing pistils.

- Figure 1 S4: This is an interesting finding and a clever analysis. However, more details on the statistical model used are necessary. For example, how was non-independence between multiple measurements made on the same individual plant measured?

  We thank the reviewer for highlighting this point. We have added more details about the statistical model used for the non-linear regression in the figure legend and changed the model source data to account for non-independence of measurements. Because pollen germination was measured on multiple pistils from the same donor plant, while *SCR01* expression was measured once per donor, we now averaged germination values per plant before fitting the model. This ensures that each expression value is paired with a single representative germination value, avoiding pseudo-replication and more accurately reflecting the relationship between *SCR01* transcript abundance and pollen germination. These clarifications have been incorporated into a new version of Fig. 1 S4. Importantly, these changes in the model source data doenot not affect our conclusions that a compatible pollen germination phenotype requires a low *SCR01* transcript abundance.

- Figure 4: Formatting for the “*S*-locus” text should be consistent with the rest of the manuscript

  Thank you for pointing this out, the updated figure is now corrected.

- Figure 4 legend: More detail is needed to sufficiently understand this figure. Specifically, it is not clear what is plotted in the summary plots for each feature.

  We have revised Figure 4 to improve clarity: we added explicit labeling of each cluster in the summary plots and clarified the corresponding x-axis information for each feature. We also expanded the figure legend to describe in greater detail what is plotted in the summary panels, so that the figure can be interpreted independently of the main text. Following these modifications, the figure should now hopefully be clearer.
